# Evaluating Adherence to Therapeutic Drug Monitoring Guidelines for Gentamicin in Neonatal Care: A Retrospective Study at the Maternity and Children’s Hospital in Makkah

**DOI:** 10.3390/children11010100

**Published:** 2024-01-15

**Authors:** Abdullah Najeh Bajaber, Mahmoud Elrggal, Wajdi F. Organji, Mohammad Adil Sulaimani, Raed Mohammed Refai, Ashraf Alsaedi, Salwa Hashim Alzamzami, Fatimah Bakor Hawsawi, Saud Tanadhub Alnefaie, Azhar Ali Alsulaimani, Adnan Alharbi, Mohammed Alnuhait, Abdullah S. Alshammari, Abdu Aldarhami, Sharaf E. Sharaf

**Affiliations:** 1 Maternity and Children Hospital, Ministry of Health, Makkah 24246, Saudi Arabia; anbajaber@moh.gov.sa (A.N.B.); worganji@moh.gov.sa (W.F.O.); mosulaimani@moh.gov.sa (M.A.S.); rrefai@moh.gov.sa (R.M.R.); asaalsaedi@moh.gov.sa (A.A.); shalzamzami@moh.gov.sa (S.H.A.); fbhawsawi@moh.gov.sa (F.B.H.); stalnefaie@moh.gov.sa (S.T.A.); azharalsulaimani@gmail.com (A.A.A.); 2 Pharmacology & Toxicology Department, Faculty of Medicine, Al Qunfudah, Umm Al-Qura University, Makkah 21961, Saudi Arabia; 3 Pharmaceutical Practices Department, College of Pharmacy, Umm Al-Qura University, Makkah 24382, Saudi Arabia; assharbi@uqu.edu.sa (A.A.); manuhait@uqu.edu.sa (M.A.); asshammari@uqu.edu.sa (A.S.A.); 4 Department of Medical Microbiology, Faculty of Medicine, Al Qunfudah, Umm Al-Qura University, Makkah 21961, Saudi Arabia; ahdarhami@uqu.edu.sa; 5 Pharmaceutical Sciences Department, College of Pharmacy, Umm Al-Qura University, Makkah 24382, Saudi Arabia; sesharaf@uqu.edu.sa

**Keywords:** gentamicin, neonatal care, therapeutic drug monitoring (TDM), respiratory distress

## Abstract

In this study, we assess healthcare providers’ adherence to therapeutic drug monitoring (TDM) guidelines for gentamicin in neonates. Conducted at the Maternity and Children’s Hospital in Makkah, Saudi Arabia, from July 2020 to July 2022, it retrospectively analyzed the compliance of healthcare workers in managing neonates treated with gentamicin. Covering 410 neonates, primarily diagnosed with respiratory distress (56%) and sepsis (32%), the study revealed that while a majority of trough and peak levels conformed to guidelines, substantial deviations were noted in cases of respiratory distress. This underlines the necessity for targeted TDM strategies, particularly in managing respiratory distress in neonates, to ensure optimal treatment efficacy and safety. The findings urge stringent compliance with TDM guidelines, emphasizing personalized approaches in neonatal gentamicin therapy for improved healthcare outcomes.

## 1. Introduction

Gentamicin, a crucial aminoglycoside antibiotic, is extensively used in neonatal care for its effectiveness against severe bacterial infections, particularly those caused by Gram-negative bacteria [1]. This antibiotic is indispensable in treating neonates, a population vulnerable to bacterial infections due to their immature immune systems. However, the therapeutic efficacy of gentamicin is counterbalanced by its narrow therapeutic window, demanding precise dosing to avoid potential nephrotoxicity and ototoxicity, which are significant concerns among this group of patients [2,3].

The pharmacokinetics of gentamicin carries great challenges among neonates, which is mainly due to the variability influenced by factors like renal immaturity, body weight, and fluid volume changes [4]. Such variability requires personalized dosing for each patient to administer accurate dosing. The variability in gentamicin dosing and monitoring practices across neonatal units underscores the critical need for standardized dosing guidelines [5]. The need for standardized practice is crucial for optimizing therapeutic efficacy and ensuring patient safety [6].

Therapeutic drug monitoring (TDM) is vital in neonatal care for the administration of aminoglycosides, particularly gentamicin, due to the significant pharmacokinetic variability in neonates. TDM enables standardized medication management, ensuring safety and efficacy tailored to individual needs. Previous research emphasized the need for precise gentamicin dosing in newborns to minimize adverse effects [1,2,7]. Dosing strategies based on TDM and its practical application in neonatal intensive care were first introduced in 2005 [3] and tested in 2007 [4]. Valitalo et al. provided the first TDM dosing guidelines and highlighted variations in gentamicin dosing and monitoring, further underlining the importance of TDM in standardizing neonatal care practices [5].

In 2019, the Ministry of Health (MOH) in Saudi Arabia developed a standardized protocol for TDM monitoring of vancomycin and aminoglycosides in the pediatric and neonate population [8]. The protocol advocates for monitoring of serum drug concentrations, enabling tailored medication management that accounts for the unique pharmacokinetic challenges in neonates, such as renal immaturity and fluctuating body weight. By following this approach, the protocol aims to optimize the therapeutic efficacy of gentamicin and plays a crucial role in minimizing risks of adverse drug reactions like nephrotoxicity and ototoxicity. This aligns seamlessly with the objective of enhancing patient safety in neonatal care, ensuring that the delicate balance between efficacy and safety is maintained in the administration of this vital antibiotic. Adhering to TDM guidelines is essential to balance these concerns, aiming to harness the therapeutic benefits of gentamicin while justifying its associated risks [9].

Despite the critical importance of TDM in neonatal gentamicin therapy, studies indicate a concerning discrepancy between the established guidelines and their actual implementation in clinical practice [10]. This gap raises questions in relation to the effectiveness of current practices and their implications for patient outcomes.

The present study, conducted at the Maternity and Children’s Hospital in Makkah, aims to evaluate the compliance of healthcare workers with the nationally established TDM guidelines for gentamicin in neonates, focusing on key aspects such as dosing intervals, serum concentration measurements, and dosage adjustments. Through this evaluation, the study also aims to shed light on the importance of dose optimizations [11] of antibiotics and to shed light on the real-world application of TDM protocols, to identify potential areas in neonatal care where improvement is required [12,13].

This study carries a great and promising potential to enhance the understanding of TDM practices for gentamicin therapy among neonates, which is a very essential step to achieving optimum patient care and improved clinical outcomes.

## 2. Methods

### 2.1. Study Design and Setting

This retrospective study, undertaken at the Maternity and Children’s Hospital in Makkah, Saudi Arabia, spanned from July 2020 to July 2022. It aimed to evaluate the adherence of healthcare workers to the MOH therapeutic drug monitoring (TDM) 2019 guidelines for gentamicin among neonatal patients [8]. A retrospective design for this study was selected to allow a comprehensive review of existing practices without the need for intervention. The timeframe was chosen to encompass a significant period post-implementation of healthcare guidelines, thereby providing a robust data set reflective of the healthcare practices.

### 2.2. Participants

*Inclusion Criteria:* The study targets neonates who were administered gentamicin therapy. Participants were aged from 1 day to 1 month. To be considered for inclusion, neonates needed to have complete medical records that detail demographic data, precise information on gentamicin dosing, thorough therapeutic drug monitoring (TDM) data, and documented clinical outcomes. This approach is designed to ensure a comprehensive evaluation of gentamicin’s impact within this age group, considering the necessary therapeutic and outcome-related information.

*Exclusion Criteria:* Neonates were excluded from the study if they exhibited any renal impairment, as gentamicin’s pharmacokinetics are significantly altered in such conditions, and we did not wish to have any confounding factor that would shift the study away from its main objective. Additionally, cases where gentamicin sampling errors occurred, or where therapy was discontinued before the administration of a second dose, were omitted to maintain data integrity. Incomplete medical records also warranted exclusion, ensuring that the study’s findings were based on complete and accurate data sets, as per the Ministry of Health guidelines. Consideration was also given to exclude neonates on medications that could interact with gentamicin, or with pre-existing conditions such as liver dysfunction or other congenital anomalies influencing gentamicin’s efficacy. These conditions might influence the drug’s absorption, metabolism, or excretion, leading to atypical therapeutic responses or adverse effects, which could skew the study’s results.

### 2.3. Study Procedure

At the Maternity and Children’s Hospital in Makkah, MCH, Saudi Arabia, a data collection form was designed and used for all gentamicin prescribing and monitoring activity on neonatal patients. The study duration for sample collection and consideration was from July 2020 to July 2022. The following information was collected: file number, age, gender, vital signs, indication, bacterial culture (type of infection), initial dose, trough and peak levels, renal function tests as serum creatinine, blood urea nitrogen (BUN), liver functions as AST, ALT, duration of hospitalization, and adverse drug reaction (ADR). Creatinine clearance was obtained using the Schwartz formula CrCl = K×ht (cm) *88.4/SCr (micromole/L), where height (ht) is patient height and K = 0.33. Creatinine clearance was taken from the (CareWare) system.

The level of compliance with TDM guidelines for gentamicin in neonates was determined by measuring the initial dose, trough and peak levels, hospitalization, and adverse drug response (ADR). According to Saudi MOH regulation, the starting dose for neonates and infants < 2 months of age is based on the gestational age and postnatal age which is categorized as follows: the initial dose for neonates < 30 weeks is 5 (mg/kg) q48h from 0–14 days; the dose for neonates <30 weeks is 5 (mg/kg) q36h after 14 days; the initial dose for neonates between 30–34 weeks is 5 (mg/kg) q36h from 0–10 days; the dose for neonates between 30–34 weeks is 5 (mg/kg) q36h after 10 days; the initial dose for neonates ≥ 35 weeks is 4 (mg/kg) q24h between 0–7 days; and the dose for neonates ≥ 35 weeks is 5 (mg/kg) q24h for more than 7 days.

A trough level should be acquired immediately prior to the administration of the third dose, according to Saudi MOH policy, and after 30 min of the completion of the third dosage infusion, the physician or pharmacist should attain a peak level. The gentamicin target trough and peak were categorized based on the type of illness. In the case of a serious illness, the target trough is less than 2 mg/L, and the target peak is between 6 and 8 mg/L. In the case of a serious illness, the target trough is less than 2 mg/L, and the target peak is between 8 and 10 mg/L. In the case of a urinary tract infection, the target trough is less than 2 mg/L, and the target peak is between 4 and 6 mg/m. In the case of synergy against Gram-positive bacteria, the target trough is less than 2 mg/L, and the target peak is between 3 and 5 mg/mL.

The next critical step was to guarantee that healthcare professionals followed TDM guidelines based on the trough and peak results. For example, if the trough is 2 mg/L and the peak is 3 to 10 mg/L, this indicates that the trough and peak are within the target. Thus, the response here, according to the MOH protocol, is to continue the same regimen and examine again in 3–4 days. When the trough is >2 mg/L and the peak is any result, the trough is considered high. Thus, according to the MOH procedure, defer the next dose and retest the level in 24 h. If indeed the result is less than 2 mg/L, continue the regimen with a less frequent interval (e.g., Q8 to Q12) and then reassess the trough and peak around the third dose.

When the trough is less than 2 mg/L and the peak is greater than 10 mg/L, the peak is considered high. Thus, according to the MOH’s TDM policy, the action here is to reduce the dose by 25% and keep the same interval, and then reassess the trough and peak around the third dose. Serum creatinine must be monitored every other day while being on aminoglycoside therapy, and the CBC should be examined twice weekly. 

Due to the prevalence of COVID-19, health precautionary protocols and procedures were implemented inside the Maternity and Children’s Hospital in Makkah, Saudi Arabia (including wearing a face mask, social distancing, and washing hands with soap, hand disinfectant, and anti-bacterial alcohol gel, as well as spraying all tools and materials that were used in the research with isopropyl alcohol 70 percent spray). When addressing patients, the principal investigator and other data collectors were following all medical cautious guidelines and procedures, ensuring that they and the participants were safe throughout the data collecting phase, which lasted no more than 20–30 min.

### 2.4. Data Collection and Analysis

Data collection involved a retrospective review of electronic medical records. Key data points included patient demographics, dosing information, TDM data (including peak and trough serum concentration measurements), and clinical outcomes. The data were specifically reviewed for compliance with dosing intervals, serum concentration measurements, and adjustments based on TDM results, as these are crucial for effective gentamicin therapy in neonates [14,15,16,17]. Descriptive statistics, including means, standard deviations, and percentages, were utilized to summarize the demographic data, clinical characteristics, and TDM parameters of the neonatal patients. Descriptive statistics were used to analyze the data, and a Chi-square test was used for comparing categorical nominal data. To assess the compliance of TDM parameters with the MOH guidelines, we compared actual gentamicin dosing, trough and peak levels, and renal and hepatic profiles against the expected values outlined in the guidelines. This comparison was conducted using Chi-square tests for categorical variables and t-tests for continuous variables, allowing us to evaluate the significance of deviations from the guidelines. The threshold for statistical significance was set at a *p*-value of less than 0.05. All analyses were conducted using the statistical software IBM SPSS v26.

### 2.5. Sample Size Estimation

Based on an estimation of a total of 1 million in-patient admissions at the MOH, SA in the year 2019, which covers the number of neonates admitted to MOH hospitals and who received gentamycin treatment [18,19], with a margin of error of 5% and a confidence interval (CI) of 0.95, a sample size of 384 patients was required to accomplish the needed CI.

### 2.6. Therapeutic Drug Monitoring (TDM) Protocol

The TDM protocol adhered to the guidelines provided by the Saudi MOH [8]. It emphasized the importance of accurate dosing and monitoring, given the variable pharmacokinetics of gentamicin in neonates, as outlined in previous studies [3,4,12]. The protocol was designed to optimize gentamicin efficacy and minimize toxicity risks, a balancing act that is crucial in neonatal care [17,20].

### 2.7. Adverse Drug Reaction Monitoring

For monitoring adverse drug reactions (ADRs), measuring creatinine clearance is integral as it serves as a key indicator of nephrotoxicity. This is particularly crucial in the context of gentamicin therapy, as gentamicin can impair renal function, especially in vulnerable neonatal populations. Assessing creatinine clearance allows for the timely identification of any detrimental impact on the kidneys. Concurrently, monitoring liver enzymes such as aspartate aminotransferase (AST) and alanine aminotransferase (ALT) is essential. These enzymes act as biomarkers for liver health, with elevated levels potentially indicating hepatotoxic effects of the drug. By tracking these specific parameters, we aim to promptly detect and manage organ-specific toxicities, thereby ensuring safer and more effective use of gentamicin in neonates.

### 2.8. Ethical Considerations

Ethical approval for the study was secured from the MOH, ensuring that all research activities followed ethical standards for research involving human subjects. Data confidentiality and patient privacy were rigorously maintained throughout the study. IRB number: H-02-K-076-0122-660.

## 3. Results

The study included a total of 410 neonates who received gentamicin therapy. The results (Table 1) indicate that 54% were males. The gestational age was mostly term neonates, comprising around 86% of the sample, with only 3% of the sample being very premature patients, and 11% late preterm (premature). The main diagnosis for the included patients was either respiratory distress syndrome 228 (56%) or sepsis 131 (32%) (Table 1).

For TDM parameters, the mean trough level was 1.2 mcg/mL ± 1, while the mean peak level was 4.5 mcg/mL ± 3. Only 14% of the trough level lies out of the reference range (RR), while only 5% of the peak level lies out of the reference range (RR). The mean value of the initial dose was 4 mg Q48h ± 0.7, 6 mg Q36h ± 1, and 11 mg Q24h ± 2.5 (Table 2). 

**Figure 1:**
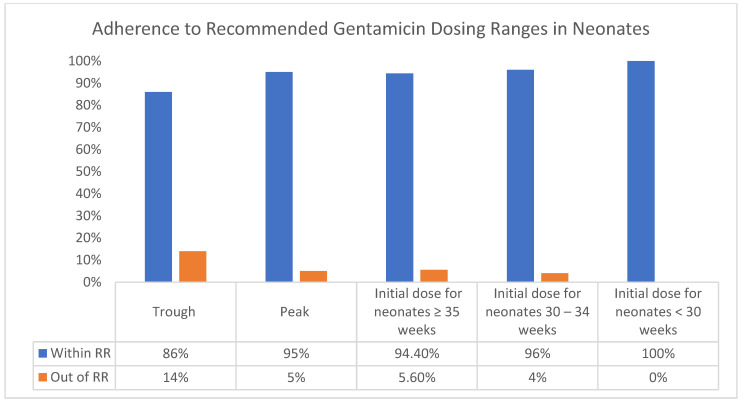
Sample percentage in and out of the reference range.

Trough levels were within the recommended range (RR) 86.1% of the time, and peak levels were even more compliant, with 95.1% within RR. Initial dosing for neonates ≥ 35 weeks was within RR in 94.4% of cases, slightly lower than the 96.6% adherence for neonates between 30–34 weeks. Remarkably, initial dosing for neonates < 30 weeks showed a 100% adherence to RR, suggesting an especially careful dosing approach for the most vulnerable, very young neonates (Figure 1).

The difference between the expected and actual trough levels was significant among neonates who had respiratory distress *p* < 0.001, while it was not significant among neonates who had sepsis and other diseases. No statistical difference was found in the peak level across diseases. This might be due to physicians prescribing smaller doses so that the peak value is not exceeded. Also, although the initial dose was statistically significantly different between the expected and actual dose, *p* < 0.001, it is not of clinical significance (Table 3).

In Table 4, the results indicate a discrepancy in the TDM parameter for trough levels in neonates at or above 35 weeks, with actual values at 299 compared to the expected 336.3, a variance with high statistical significance (*p* < 0.001). Conversely, for neonates between 30–34 weeks, and those under 30 weeks, the actual and expected values for trough, peak, and dose closely align, with *p*-values of 0.86423, 0.12381, and 0.44673, respectively, indicating no significant difference. Specifically, actual trough levels were 43 versus an expected 42.75 for the 30–34 weeks group, and 11 compared to an expected 10.45 for the under 30 weeks group. 

The mean value of alanine transaminase (ALT) was 41 U/L ± 33, with 92.5% of it lying within the reference range (RR). Moreover, the mean value of aspartate transaminase (AST) was 24 U/L ± 16, with only 6.4% lying out of the reference range (RR). The mean value of creatinine clearance was 54 mL/min ± 17, and the results for 83.6% of participants lie within the reference range (Table 5).

Table 6 highlights the adjustments made in study variables. The adherence to the recommended trough levels was observed to be higher in males (89%) compared to females (83%), and this adherence increased with gestational age, showing a 100% adherence in neonates under 30 weeks. Similarly, peak level adherence was high across all demographics, with perfect adherence in the 30–34 weeks and <30 weeks gestational age groups. The initial dose adherence also showed a pattern of increased compliance with decreasing gestational age. Regarding liver function tests, both ALT and AST levels were within the reference range for a high percentage of neonates across all demographics, with a particularly high adherence in neonates with gestational ages of 30–34 weeks and <30 weeks. Creatinine clearance (CrCl) levels varied significantly with gestational age, showing the highest adherence (92%) in neonates ≥ 35 weeks and a markedly lower adherence in the 30–34 weeks group (37.8%), while there was no normal CrCl in the <30 weeks gestational age group.

## 4. Discussion

The current study evaluated the adherence of healthcare workers to therapeutic drug monitoring (TDM) guidelines for gentamicin among neonates at the Maternity and Children’s Hospital in Makkah. Our findings revealed a mixed degree of compliance with the guidelines. Most dosing intervals and serum concentration measurements were found within the recommended reference range. However, a notable percentage of trough and peak levels were outside the recommended range, particularly in neonates with respiratory distress. These variations in TDM adherence echo the diverse outcomes reported in existing literature, highlighting the complexity of gentamicin therapy in neonatal care.

Current findings of TDM adherence in neonates for gentamicin therapy are in alignment with multiple existing studies, illustrating a diverse range of outcomes and practices. For instance, O’Connor et al. (2021) found that adherence to TDM guidelines does not significantly influence clinical outcomes in neonates with normal renal function [21]. In contrast, Fonzo-Christe et al. (2014) reported improved clinical outcomes following adherence [22].

Ismail et al. (1997) reported that TDM adherence to gentamicin led to an improved administration and shorter therapy duration in neonates [23]. Flint and Allegaert (2020), however, noted that TDM guidelines in critically ill neonates often resulted in a substantial proportion of subtherapeutic and supratherapeutic concentrations, indicating a need for more individualized TDM strategies [24]. These studies collectively highlight the complexity and necessity of tailored TDM in neonatal care, especially given the variability in guideline adherence.

Our findings suggest a need for more robust implementation and monitoring of TDM guidelines in neonatal gentamicin therapy. This suggestion is in alignment with ongoing strategies to optimize the rational use of antibiotics through multidisciplinary programs and various techniques [25].

The significance of the current investigation lies in its robust sample size and detailed data analysis, offering comprehensive insights into TDM practices. However, the study is not without limitations. Its retrospective nature, while beneficial in some respects, limits the ability to establish causality between TDM compliance and clinical outcomes. The study’s focus on a single healthcare facility may also limit the generalizability of the findings to other settings. Furthermore, as with any study reliant on hospital records, there is a potential for missing or incomplete data, which might affect the accuracy of the conclusions drawn.

One limitation of our study is the lack of interprofessional involvement in the handling of gentamicin, similar to the approach advocated by Schmid et al. in their 2023 study on therapeutic drug monitoring of carbapenems [26]. They emphasized the improved ICU care and guideline adherence resulting from interprofessional collaboration in drug monitoring. This underscores the potential benefits of incorporating a multidisciplinary approach in TDM for gentamicin in neonates, which may enhance guideline adherence and patient care quality.

This study opens several avenues for future research. Prospective studies are needed to establish a causal relationship between TDM compliance and clinical outcomes in neonates. Future studies should also explore the factors contributing to non-compliance with TDM guidelines and discuss strategies to improve adherence.

Future research should also explore the impact of personalized TDM strategies tailored to individual patient characteristics, such as gestational age and coexisting health conditions, on the efficacy and safety of gentamicin therapy. Investigating the role of advanced pharmacokinetic modeling and simulation tools in optimizing gentamicin dosing in neonates could be another promising direction. Overall, these future research efforts should aim not only to enhance the understanding of TDM in neonatal care but also to directly translate into improved clinical practices and outcomes for this vulnerable patient population.

## 5. Conclusions

The study’s findings highlight the critical need for more stringent adherence to TDM guidelines in neonatal gentamicin therapy. Despite compliance in certain areas, the variability in adherence, especially in cases of respiratory distress, calls for a more individualized and careful approach to TDM in neonates. This research underscores the complexity of optimizing gentamicin therapy and the importance of regular monitoring to ensure efficacy and minimize risks, paving the way for enhanced patient care and improved clinical outcomes in neonatal populations.

## Figures and Tables

**Table 1:** Demographics.

Variables	N%
Gender	
Male	221 (54%)
Female	189 (46%)
Total	410 (100%)
Gestational Age	
≥35 weeks	354 (86%)
30–34 weeks	45 (11%)
<30 weeks	11 (3%)
Total	410 (100%)
Diagnosis	
Respiratory distress syndrome	228 (56%)
Sepsis	131 (32%)
Other	51 (12%)
Total	410 (100%)

**Table 2:** TDM parameters.

Variables	Mean ± SD Unit	Reference Range N%	Within RR N%	Out of RR N%
Trough mcg/mL	1.2 mcg/mL ± 1	0–2 mcg/mL	353 (86.1%)	57 (13.9%)
Peak mcg/mL	4.5 mcg/mL ± 3	0–10 mcg/mL	390 (95.1%)	20 (4.9%)
**Initial Dose mg/kg**	**Mean ± SD Unit**	**Reference Range N%**	**Within RR N%**	**Out of RR N%**
≥35 weeks	11 mg Q24h ± 2.5	5 mg/kg Q24h	334 (94.4%)	20 (5.6%)
30–34 weeks	6 mg Q36h ± 1	5 mg/kg Q36h	43 (95.6%)	2 (4.4%)
<30 weeks	4 mg Q48h ± 0.7	5 mg/kg Q48h	11 (100%)	None

**Table 3:** Difference between expected and actual results for TDM parameters according to diagnosis.

Variable	Respiratory Distress			Sepsis			Others		
	Expected	Actual	*p*-Value	Expected	Actual	*p*-Value	Expected	Actual	*p*-Value
Trough	216.6	191	<0.001	216.6	218	0.670	216.6	216	0.855
Peak	124.5	124	0.856	124.45	128	0.154	124.45	127	0.306
Initial dose	48.5	38	<0.001	48.45	44	0.004	48.45	45	0.026

**Table 4:** Difference between expected and actual results for TDM parameters according to gestational age.

Variable	≥35 Weeks			30–34 Weeks			<0 Weeks		
	Expected	Actual	*p*-Value	Expected	Actual	*p*-Value	Expected	Actual	*p*-Value
Trough	336.3	299	<0.001	42.75	43	0.864	10.45	11	0.446
Peak	336.3	334	0.574	42.75	45	0.123	10.45	11	0.446
Dose	336.3	334	0.574	42.75	43	0.864	10.45	11	0.446

**Table 5:** Adverse drug reaction (ADR) and toxicity parameters.

Variables	Mean ± SD Unit	Reference Range N%	Within RR N%	Out of RR N%
ALT (U/L)	41 ± 33	14–84	379 (92.5%)	31 (7.5%)
AST (U/L)	24 ± 16	8–33	384 (93.6%)	26 (6.4%)
Creatinine clearance (mL/min)	54 ± 17	40–100	343 (83.6%)	67 (16.4%)
Hospitalization (days)	21 ± 16	7–14 days	179 (43.7%)	231 (56.3%)

**Table 6:** Adjustment of study variables according to different demographics.

Normal Variables	Gender		Gestational Age			Disease		
	Male	Female	≥35 Weeks	30–34 Weeks	<30 Weeks	Respiratory Distress Syndrome	Sepsis	Other
Normal trough	89%	83%	84.5%	95.6%	100%	84%	95%	75%
Normal peak	94.2%	96.3%	94.3%	100%	100%	96%	98%	86%
Initial dose	96%	93%	94.4%	96%	100%	95%	97%	88%
Normal ALT	92.3%	92.6%	91.3%	100%	100%	90%	96%	96%
Normal AST	94.6%	92.6%	94.1%	91.1%	91%	94%	95%	92%
Normal CrCl	83.7%	83.6%	92%	37.8%	0%	94%	67%	80%

CrCl: creatinine clearance, ALT: alanine aminotransferase, AST: aspartate aminotransferase.

## Data Availability

The data presented in this study are available on request from the corresponding author. The data are not publicly available due to privacy and ethical.

## References

[1] Chattopadhyay B. (2002). Newborns and gentamicin—How much and how often?. J. Antimicrob. Chemother..

[2] Kahlmeter G., Dahlager J.I. (1984). Aminoglycoside toxicity—A review of clinical studies published between 1975 and 1982. J. Antimicrob. Chemother..

[3] De Hoog M., Mouton J.W., van den Anker J.N. (2005). New dosing strategies for antibacterial agents in the neonate. Semin. Fetal Neonatal Med..

[4] Testa M., Fanos V., Martinelli V., Stronati M., Mussap M., Del Zompo M. (2007). Therapeutic Drug Monitoring of Gentamicin in Neonatal Intensive Care Unit: Experience in 68 Newborns. J. Chemother..

[5] Valitalo P.A., van den Anker J.N., Allegaert K., de Cock R.F., de Hoog M., Simons S.H., Mouton J.W., Knibbe C.A. (2015). Novel model-based dosing guidelines for gentamicin and tobramycin in preterm and term neonates. J. Antimicrob. Chemother..

[6] Saddi V., Preddy J., Dalton S., Connors J., Patterson S. (2017). Variation in Gentamicin Dosing and Monitoring in Pediatric Units across New South Wales. Pediatr. Qual. Saf..

[7] Fanos V., Cuzzolin L., Atzei A., Testa M. (2007). Antibiotics and Antifungals in Neonatal Intensive Care Units: A Review. J. Chemother..

[8] Mohzeri Y. (2019). Therapeutic Drug Monitoring (TDM) Protocol for Pediatric.

[9] Fanos V., Cataldi L. (1999). Antibacterial-Induced Nephrotoxicity in the Newborn. Drug Saf..

[10] National Institute for Health and Clinical Excellence Great Britain (2012). Antibiotics for Early-Onset Neonatal Infection: Antibiotics for the Prevention and Treatment of Early-Onset Neonatal Infection.

[11] Elrggal M.E., Haseeb A., AlGethamy M., Ahsan U., Saleem Z., Althaqafi A.S., Alshuail S.S., Alsiddiqi Z.A., Iqbal M.S., Alzahrani A.F. (2023). Dose optimization of vancomycin in obese patients: A systematic review. Front. Pharmacol..

[12] Pacifici G.M. (2015). Clinical pharmacology of gentamicin in neonates: Regimen, toxicology, and pharmacokinetics. Med. Express.

[13] Berkovitch M., Goldman M., Silverman R., Chen-Levi Z., Greenberg R., Marcus O., Lahat E. (2000). Therapeutic drug monitoring of once daily gentamicin in serum and saliva of children. Eur. J. Pediatr..

[14] Murgitroyd E., Farquharson S., Poole N. (2015). Non-compliance in gentamicin prescribing and administration: A patient safety issue. Schumacher U, editor. Cogent Med..

[15] Antolik T.L., Cunningham K.J., Alabsi S., Reimer R.A. (2017). Empirical gentamicin dosing based on serum creatinine levels in premature and term neonates. Am. J. Health Syst. Pharm..

[16] Mustafa S., Noor S., Razman R., Wan Yusuf W.N. (2022). Monitoring of gentamicin blood level in one-week-of-life neonates admitted to a special care nursery ward. Gulhane Med. J..

[17] Gross A.S. (2001). Best practice in therapeutic drug monitoring. Br. J. Clin. Pharmacol..

[18] SurveyMonkey (2019). Sample Size Calculator. https://www.surveymonkey.com/mp/sample-size-calculator/.

[19] (2019). Statistical Yearbook Ministry of Health. https://www.moh.gov.sa/en/Ministry/Statistics/book/Pages/default.aspx.

[20] Pereboom M.J., Mulder I.L., Verweij S., van der Hoeven R.L., Becker M. (2019). A clinical decision support system to improve adequate dosing of gentamicin and vancomycin. Int. J. Med. Inform..

[21] O’Connor K., Davies M., Koorts P., Cartwright D., Whitfield K. (2021). Gentamicin Dosing in Neonates with Normal Renal Function: Trough and Peak Levels. Eur. J. Drug Metab. Pharmacokinet..

[22] Fonzo-Christe C., Guignard B., Zaugg C., Coehlo A., Posfay-Barbe K., Gervaix A., Desmeules J., Rollason V., Combescure C., Corbelli R. (2014). Impact of Clinical Decision Support Guidelines on Therapeutic Drug Monitoring of Gentamicin in Newborns. Ther. Drug Monit..

[23] Ismail R., Haq A., Azman M., Rahman A. (1997). Therapeutic drug monitoring of gentamicin: A 6-year follow-up audit. J. Clin. Pharm. Ther..

[24] Flint R., Allegaert K. (2020). Target Drug Exposure Attainment in Children: How to Get from Better to Best. Paediatr. Drugs.

[25] Haseeb A., Faidah H.S., Al-Gethamy M., Iqbal M.S., Barnawi A.M., Elahe S.S., Bukhari D.N., Noor Al-Sulaimani T.M., Fadaaq M., Alghamdi S. (2021). Evaluation of a Multidisciplinary Antimicrobial Stewardship Program in a Saudi Critical Care Unit: A Quasi-Experimental Study. Front. Pharmacol..

[26] Schmid S., Koch C., Zimmermann K., Buttenschoen J., Mehrl A., Pavel V., Schlosser-Hupf S., Fleischmann D., Krohn A., Schilling T. (2023). Interprofessional Therapeutic Drug Monitoring of Carbapenems Improves ICU Care and Guideline Adherence in Acute-on-Chronic Liver Failure. Antibiotics.

